# Current Challenges in Pancreas and Islet Transplantation: A Scoping Review

**DOI:** 10.3390/biomedicines12122853

**Published:** 2024-12-15

**Authors:** Velimir Altabas, Tomislav Bulum

**Affiliations:** 1 Department of Endocrinology, Diabetes and Metabolic Diseases Mladen Sekso, Sestre Milosrdnice University Hospital Center, 10000 Zagreb, Croatia; 2 School of Medicine, University of Zagreb, 10000 Zagreb, Croatia; 3 Vuk Vrhovac University Clinic for Diabetes, Endocrinology and Metabolic Diseases, Merkur University Hospital, 10000 Zagreb, Croatia

**Keywords:** type 1 diabetes, islet transplantation, pancreas, β-cell

## Abstract

Type 1 diabetes mellitus is an autoimmune condition characterized by the destruction of pancreatic β-cells, necessitating insulin therapy to prevent life-threatening complications such as diabetic ketoacidosis. Despite advancements in glucose monitoring and pharmacological treatments, managing this disease remains challenging, often leading to long-term complications and psychological burdens, including diabetes distress. Advanced treatment options, such as whole-pancreas transplantation and islet transplantation, aim to restore insulin production and improve glucose control in selected patients with diabetes. The risk of transplant rejection necessitates immunosuppressive therapy, which increases susceptibility to infections and other adverse effects. Additionally, surgical complications, including infection and bleeding, are significant concerns, particularly for whole-pancreas transplantation. Recently, stem cell-derived therapies for type 1 diabetes have emerged as a promising alternative, offering potential solutions to overcome the limitations of formerly established transplantation methods. The purpose of this scoping review was to: (1) summarize the current evidence on achieved insulin independence following various transplantation methods of insulin-producing cells in patients with type 1 diabetes; (2) compare insulin independence rates among whole-pancreas transplantation, islet cell transplantation, and stem cell transplantation; and (3) identify limitations, challenges and potential future directions associated with these techniques. We systematically searched three databases (PubMed, Scopus, and Web of Science) from inception to November 2024, focusing on English-language, peer-reviewed clinical studies. The search terms used were ‘transplantation’ AND ‘type 1 diabetes’ AND ‘insulin independence’. Studies were included if they reported on achieved insulin independence, involved more than 10 patients with type 1 diabetes, and had a mean follow-up period of at least one year. Reviewers screened citations and extracted data on transplant type, study population size, follow-up duration, and insulin independence rates. We identified 1380 papers, and after removing duplicates, 705 papers remained for title and abstract screening. A total of 139 English-language papers were retrieved for full-text review, of which 48 studies were included in this review. The findings of this scoping review indicate a growing body of literature on transplantation therapy for type 1 diabetes. However, significant limitations and challenges, like insufficient rates of achieved insulin independence, risks related to immunosuppression, malignant diseases, and ethical issues remain with each of the established techniques, highlighting the need for innovative approaches such as stem cell-derived islet transplantation to promote β-cell regeneration and protection.

## 1. Introduction

Diabetes mellitus encompasses a group of chronic and progressive metabolic disorders with various causes, distinct pathophysiological mechanisms, and treatment options. The main pathophysiological disturbances involve impaired carbohydrate metabolism, leading to reduced glucose utilization in peripheral tissues, along with inappropriate gluconeogenesis and glycogenolysis, resulting in hyperglycemia. Although there are different subtypes of diabetes, reflecting the heterogeneity of the condition, the diagnostic criteria primarily rely on elevated blood glucose levels and increased A1c as a surrogate marker [1,2].

The hallmark of diabetes is chronic hyperglycemia, which is associated with a range of acute and chronic complications. Despite modern treatment options, these complications remain a significant cause of disability and premature death in both developed and developing countries. Diabetes, as a group of heterogeneous disorders, can be classified into distinct types, including type 1, type 2, gestational diabetes mellitus, and other specific types caused by factors such as genetic mutations, exocrine pancreatic disorders, endocrine diseases, or medications [2].

The most common type of diabetes is type 2 (T2DM), followed by type 1 (T1DM) and gestational diabetes mellitus (GDM). T2DM accounts for over 90% of cases worldwide, while GDM is generally limited to pregnancy. T1DM, although less common, affects more than 9 million people globally. It poses a substantial burden due to its treatment complexity and the risk of both acute and chronic complications [2,3].

T1DM results from the autoimmune destruction of pancreatic β-cells, leading to insulin deficiency. For patients with a substantial loss of β-cell number and function, insulin treatment is necessary to prevent acute, life-threatening complications such as diabetic ketoacidosis [4]. Over time, if blood glucose control is not optimal, microvascular and macrovascular complications may develop. Microvascular complications, such as diabetic retinopathy, neuropathy, and nephropathy, are now the leading causes of blindness, non-traumatic limb amputations, and the need for kidney replacement therapy in developed countries. In addition, macrovascular complications, including myocardial infarction and stroke, are significant contributors to disability and premature death in these patients [5].

**Figure 1:**
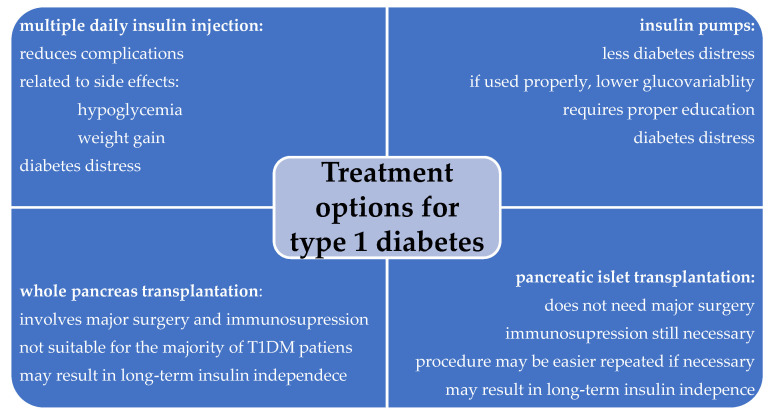
Currently available treatment options for type 1 diabetes.

Despite recent advancements in the treatment of clinical and even preclinical T1DM—including the development of insulin analogues, automated insulin pumps, continuous glucose monitoring systems, closed-loop insulin delivery systems, and teplizumab (a humanized anti-CD3 monoclonal antibody approved for delaying the onset of clinically apparent T1DM)—managing the condition remains a significant burden for patients, requiring constant blood sugar monitoring and adjustments [6,7]. Figure 1 illustrates the currently available treatment options for clinically apparent T1DM. In addition, the constant care required to manage diabetes can lead to psychological challenges, known as diabetes distress. This refers to the emotional strain caused by living with the disease and the burden of continuous daily self-management, which can hinder optimal glucose regulation and clinical outcomes, and further reduce the quality of life [8].

Pancreas transplantation (PTx) and pancreatic islet transplantation (IT) offer advanced approaches for treating and potentially curing diabetes [9]. These procedures are currently reserved mainly for a specific group of diabetic patients who suffer from severe and recurrent hypoglycemic episodes while on insulin therapy. As a result, to avoid hypoglycemia, these patients may reduce or omit insulin, which prevents them from maintaining strict glycemic control. This significantly increases their risk of developing diabetic ketoacidosis as well as chronic microvascular and macrovascular complications, limiting their life expectancy and quality of life [5]. For these reasons, therapeutic approaches that aim to restore endogenous insulin production offer a promising solution [9,10].

Although these options have been established in clinical medicine for several decades, they still face significant limitations, including inconsistent success in achieving insulin independence, risks associated with immunosuppression, potential malignancies, and ethical concerns. As a result, innovative approaches such as stem cell-derived islet transplantation to promote β-cell regeneration and protection are being actively explored.

This review aims to: (1) summarize the current evidence on achieving insulin independence through various transplantation methods of insulin-producing cells in patients with type 1 diabetes; (2) compare insulin independence rates across whole-pancreas transplantation, islet cell transplantation, and stem cell transplantation; and (3) identify the limitations, challenges and potential future directions associated with these techniques.

## 2. Materials and Methods

### 2.1. Methods, Procedures, and Screening Process

**Figure 2:**
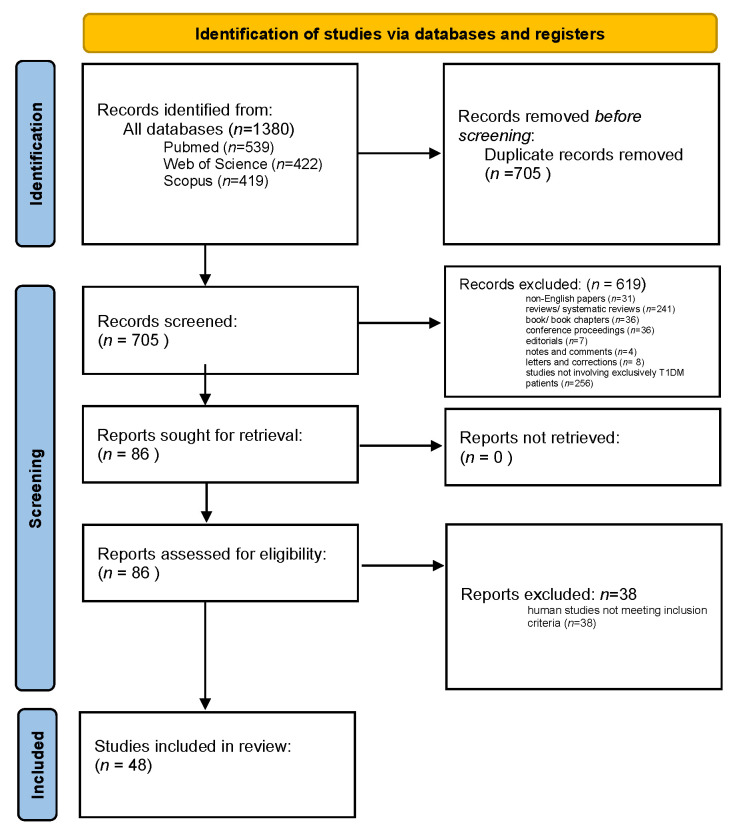
Sequence of steps involved in the collection and selection of qualified studies.

This literature review followed the guidelines of the Preferred Reporting Items for Systematic Reviews and Meta-Analyses extension for Scoping Reviews (PRISMA-ScR). The flow diagram in Figure 2 illustrates the sequence of steps involved in the collection and selection of qualified studies.

We conducted a comprehensive search for clinical studies reporting insulin independence as the ultimate goal of various pancreas transplantation techniques, including whole-pancreas transplantation, islet transplantation, and stem cell-derived therapeutic options. The search utilized the terms transplantation AND type 1 diabetes AND insulin independence and was performed across PubMed, Scopus, and Web of Science, covering studies published from inception to November 2024. Titles and abstracts were independently screened and retrieved from database searches.

The suitability of this article was then assessed according to the defined inclusion criteria. Ultimately, we received all titles and abstracts that met the criteria for inclusion in the full text.

### 2.2. Inclusion Criteria

A study was included if it reported the rates of achieved insulin independence after a mean follow-up of at least one year in a cohort of more than 10 patients with T1DM who underwent either whole-pancreas transplantation, islet transplantation, or stem cell-based therapies. This review included only articles written in English.

### 2.3. Exclusion Criteria

A paper was excluded if it lacked data or information on achieved insulin independence in the specified patient group, if the follow-up period was shorter than one year, or if the patient cohort was smaller than the prespecified size. Papers reporting outcomes in patients with other types of diabetes were also excluded, as well as preclinical studies, systematic reviews, integrative reviews, narrative reviews, editorials, commentaries, letters, notes, and case reports. All articles written in languages other than English were excluded.

## 3. Results

### Overall Search Results

A total of 1380 papers containing the search term in the title or abstract were identified across PubMed (539), Scopus (422), and Web of Science (419). After removing duplicates, 705 papers remained for further screening. Papers published in languages other than English, along with reviews, preclinical studies, editorials, commentaries, letters, notes, and corrections, were excluded, leaving 86 papers for full-text review. From these, case reports, small case series involving fewer than 10 patients with type 1 diabetes, and studies with a follow-up period of less than one year were excluded. Ultimately, 48 papers were included in this review. Summaries of these studies are presented in the discussion sections of this review. The search process and results are illustrated in Figure 2.

A critical appraisal of the quality of the selected articles was conducted using the Critical Appraisal Skills Programme (CASP) checklists, which consist of 12 questions per paper. The details of these questions are described elsewhere [11]. The outcomes for each included study are presented in Table 1.

A key characteristic of these 48 studies is their small sample size: 32 studies included fewer than 50 participants, while another 12 had between 51 and 100 participants. In 14 of the 48 included studies, potential bias related to inclusion criteria was identified, such as using data from registries that may not cover the entire study population and the lack of information regarding T1DM duration. Among the seven studies reporting on AHSC transplantation, five lacked a control group of patients with newly diagnosed T1DM, which hinders the quality of these manuscripts. Finally, three studies reported a significant proportion of patients lost to follow-up.

## 4. Discussion

### 4.1. Pancreas Transplantation

PTx restores an autoregulating gland that produces endogenous insulin, responsive to normal feedback mechanisms. In PTx, the donor pancreas is implanted in an ectopic location, typically in the lower abdomen. It is usually surgically connected to the recipient’s small intestine—rarely the bladder—to drain digestive enzymes from the exocrine pancreas, minimizing surgical complications [9,60]. In the ideal scenario, PTx improves glycemic variability and eliminates hypoglycemic episodes, restores normal glucose homeostasis without insulin use, ameliorates chronic diabetic complications, and enhances both quality of life and life expectancy. Additionally, a successful PTx supports the secretion of counterregulatory hormones and restores exocrine pancreatic function [61,62].

The first PTx was performed in 1966, and since then, significant experience has been gained. Today, PTx is recognized as an established therapeutic option for patients with brittle diabetes. While more than 80% of PTx procedures are performed in patients with T1DM, PTx has increasingly been used in patients with other forms of diabetes, such as T2DM and cystic fibrosis-related diabetes [9,10].

Several strategies for PTx were developed, including simultaneous pancreas and kidney (SPK) transplants, pancreas-after-kidney (PAK) transplants, and pancreas transplants alone (PTA). In relatively rare cases, PTx is combined with lung or liver transplantation, predominantly in patients with cystic fibrosis. Pancreas transplantation has also been described in patients with post-pancreatectomy diabetes. In such cases, the pancreatic graft was able to restore both insulin production and digestive enzyme secretion [9,63,64]. Nevertheless, SPK remains the most commonly performed PTx procedure in patients with T1DM. Patients undergoing this type of surgery benefit most from PTx, as they would already need to undergo major surgery and immunosuppression for renal transplantation [63]. Conversely, patients with T1DM and renal failure who underwent SPK transplantation had better outcomes, with reduced 10- and 20-year mortality rates compared to those who received only a kidney transplant, according to a Dutch nationwide cohort study [65].

Other strategies for PTx, such as PAK and PTA, are used less frequently. PAK typically involves two independent donors and procedures: a living kidney donor, who may be related to the patient, and a separate deceased donor for the pancreas, adding complexity to the overall process. Meanwhile, PTA was traditionally reserved for patients with T1DM who had preserved kidney function. Both strategies have historically been associated with higher mortality rates compared to patients remaining on the waiting list, leading to their less frequent utilization [61,66,67].

In the past, PTx was generally regarded as a procedure associated with a high rate of early allograft loss, frequent complications, and significant morbidity. In the meantime, surgical techniques, strategies, and immunosuppression protocols for PTx have significantly evolved, with the aim of reducing surgical complications such as thrombosis, pancreatitis, infections, hemorrhage, and fistula formation, as well as minimizing graft rejection, infections and organ toxicity [68,69]. As a result, outcomes for PTx have improved significantly, with patient survival rates now ranging from 96% to 98% at one year and approximately 90% at five years across all PTx categories. In contrast, only 60% of patients with T1DM on the waiting list for SPK transplantation remain alive after 5 years [61,70].

More recent data indicate that over 80% of patients have a functioning pancreas graft for more than five years following PTx, and approximately 75% maintain function after 10 years [71]. The latest reports indicate that insulin independence rates, as a favorable metabolic outcome, are observed in over 90% of patients in the short to medium term, but these rates tend to decline during longer follow-up periods. Data on reported insulin independence rates, study population sizes, and follow-up periods are summarized in Table 2.

However, since large, high-volume centers in the US and EU have reported better outcomes compared to smaller centers, it is possible that center size, along with the year of the study and the available technological and immunosuppressive support at the time, could affect the published results [60,72].

Contemporary challenges in PTx include the limited number of suitable donors and geographic disparities in access to high-volume centers and transplantable pancreases. Currently, graft survival rates are similar between pancreases taken from donors after circulatory death and brain death, expanding the potential pool of organs available for transplantation. Previously, brain death donors were considered more favorable for graft survival. Additionally, networks like Eurotransplant and allocation systems in the US have improved access to PTx by reducing waiting times and addressing some of the geographic disparities in transplant availability. Moreover, a higher annual volume of PTx at certain centers is expected to contribute to better outcomes, although available data do not clearly define a minimum annual volume. This outcome can also be influenced by various geographical factors, as well as donor and recipient selection [63,73,74,75].

It is important to note that despite steadily improving outcomes and broader recipient selection criteria, the number of PTx procedures performed has declined in recent years. This decline can be attributed to a prolonged lack of consensus criteria, the absence of a primary referral source, limited acceptance among diabetes care professionals, and challenges related to access, education, and resources within the transplantation process. Additionally, the development of less invasive procedures, such as pancreatic islet transplantation, may have contributed to this trend [63,73,75].

### 4.2. Pancreatic Islet Transplantation

In contrast to PTx, pancreatic IT was developed as a less invasive procedure, typically involving the infusion of islet cells from a donor pancreas into the portal vein or omental vessels. The use of islet cells increased the availability of donor pancreata by allowing the utilization of organs that are not suitable for whole-organ transplantation. Major complications typically associated with surgery are largely avoided, with bleeding being the most common complication [76]. It has been used in humans since the 1980s, but results remained unsatisfactory until improvements were made in the 2000s. These advancements included better donor selection, improvements in the quantity and quality of transplanted islets, and the introduction of less toxic corticosteroid-free immunosuppressant protocols [73,77,78]. The greater availability of donor pancreases and the avoidance of major surgery have increased the number of potential recipients. As a result, unlike PTx, most procedures involve islet transplantation alone (ITA), while islet-after-kidney (IAK) transplantation is less common, and simultaneous islet-kidney (SIK) transplantation is rare [9].

Deceased donors (cadavers) are the primary source of human islets for transplantation, and procedures can be repeated by combining islets from multiple donors. The surgical procedure is considered less invasive compared to whole-organ pancreas transplantation and can be performed either via transhepatic percutaneous infusion or omental vein cannulation under radiologic guidance. The islets are infused into the portal vein system, where they become engrafted in the liver. Following a successful procedure, glucose stability is typically achieved within days to weeks, while insulin independence occurs later, once the islets are fully engrafted and revascularized in their new environment [27]. The success of IT has improved over time, with a steadily increasing proportion of patients achieving insulin independence. However, insulin independence rates remain lower than those achieved with PTx, and multiple IT procedures are sometimes required for clinical success. Notable advances in IT success became evident as early as 2012, when data from the Collaborative Islet Transplant Registry were published. However, as reported in this paper, long-term insulin independence was achieved in less than 50% of IT recipients [36].

Historically, the first larger trial reviewed that reported insulin independence rates above 50% was published in 2016. This phase III multicenter clinical trial involved 48 patients with T1DM who experienced severe hypoglycemia and received one or more islet IT procedures. The study’s primary endpoint was defined somewhat differently, as HbA1c levels below 7.0% and the absence of severe hypoglycemic events from day 28 to day 365, which was achieved by 87.5% of the participants. An insulin independence rate of 52.1 at day 365 was reported [27].

Recently, several large studies on the long-term outcomes of IT have been published. One study, involving patients from the Edmonton Centre who underwent IT, reported that insulin independence was achieved in 78.6% of recipients, with a mean duration of insulin independence of 2.1 years [12]. The same authors previously reported more favorable outcomes in patients with surviving grafts, defined as fasting plasma C-peptide levels greater than 0.1 nmol/L. In these patients, insulin independence rates reached 90%. In contrast, patients with failed graft survival had poorer outcomes, with only 53% achieving insulin independence [17]. The data on reported insulin independence rates, study population sizes, and follow-up periods are presented in Table 3.

Factors associated with favorable outcomes in IT were identified in a cohort of 398 patients with T1DM. Four key factors were determined: recipient age of 35 years or older, a total of at least 325,000 islet equivalents (IEQ) infused, induction immunosuppression with T cell depletion and/or TNF-α inhibition, and maintenance therapy with both mTOR inhibitors and calcineurin inhibitors. After 5 years, 53% of patients who met these criteria were insulin independent and exhibited significantly better blood glucose control [14].

A recent multicenter retrospective study of 1210 patients who received either IT alone or IT after kidney transplantation demonstrated a linear independent relationship between primary graft survival,—measured by the Beta 2 score (a complex index incorporating fasting blood glucose, C-peptide, HbA1c, and insulin dose as indicators of graft function)—and 5-year adverse clinical outcomes of IT [79]. These outcomes included unsuccessful IT, graft exhaustion, inadequate glucose control, and the need for exogenous insulin therapy. The study also identified other relevant factors, such as the characteristics of the recipient and transplanted islets and the transplantation strategies and immunosuppression regimens used [13].

Some clinical difficulties related to IT in the liver still need to be addressed. While the intraportal infusion site is easily accessible, islet infusion triggers a proinflammatory and procoagulatory host response, which can lead to islet loss and IT failure [80]. Therefore, alternative sites for IT are being explored, such as the bone marrow, subcutaneous tissue and the omentum, where biological scaffolds may be used to protect the transplanted islets from immune destruction [81,82]. In addition, immunosuppressant drugs like tacrolimus and cyclosporine, used for maintenance therapy, reach significantly higher concentrations in the portal circulation compared to systemic blood flow, resulting in toxic effects on the transplanted beta cells [83].

Immunosuppression remains another significant challenge for successful IT. Typically, T-cell-depleting agents, along with tumor necrosis factor-alpha (TNFα) inhibitors and anti-CD3 antibodies, are used for immunosuppression induction. Although introduced later, anakinra and etanercept used during the induction phase have been associated with more favorable outcomes in the Edmonton study [17]. Maintenance requires a two-drug regimen, with tacrolimus or ciclosporin as critical components, combined with mycophenolate mofetil. Alternatively, co-stimulation inhibitors such as efalizumab or belatacept may be used in patients prone to renal injury [9,84]. In terms of clinical outcomes, these immunosuppression protocols have resulted in improved insulin independence rates, although some patients required more than one islet transplantation to achieve clinical success [29]. As with pancreas transplantation (PTx), immunosuppression was associated with an increased risk of infections and tumorogenesis [68,69].

Lastly, the legal regulation of islet cells varies across the world. In the United States, islets cells are regulated as a manufactured biologic product and need to be administered under an Investigational New Drug Application. This led to the development and recent approval of donislecel, the first allogenic cell-based therapy for T1DM, granted by the FDA, after clinical trials showed efficacy [85]. According to data published on ClinicalTrials.gov, a Phase 3 clinical trial involving 21 patients with T1DM reported that 19 patients experienced no severe hypoglycemic events at the one-year follow-up on day 365. Additionally, 19 out of 21 patients achieved HbA1c levels of ≤6.5%, with approximately half becoming insulin-independent. It is recommended that patients undergoing this therapy take immunosuppressive medications to prevent rejection by their immune system [86]. In contrast, allogenic islet cell administration in the European Union is regulated similar to whole-organ transplantation and does not require clinical trial authorization or marketing review by a regulatory authority.

### 4.3. Islet Stem Cell Transplantation

The limited availability of pancreatic islets still poses a challenge to the further development of IT. However, modern technology enables the use of various types of stem cells. Although these cells are less differentiated than mature islet cells, they have a favorable ability to proliferate and differentiate later, making them a promising source for direct transplantation or in vitro islet farming. Autologous hematopoietic stem cells (AHSCs), human embryonic stem cells (ESCs), and induced pluripotent stem cells (iPSCs) are being investigated in this area.

While clinical experience with ESCs and iPSCs remains limited, several papers report clinically successful AHSC transplantations in patients with newly diagnosed T1DM. These cells are typically used in newly diagnosed patients with T1DM after AHSC mobilization with cyclophosphamide and granulocyte growth factor, followed by collection through apheresis procedures and immunologic conditioning using cyclophosphamide and anti-thymocyte serum or fludarabine. Typically, a single procedure is conducted [29]. The exact mechanism of ADSCs in autoimmune diseases is still under debate. Possible mechanisms include active or passive tolerance, T-regulatory cell suppression, or clonal deletion. It appears that these procedures work efficiently, at least in newly diagnosed T1DM, by prolonging the naturally occurring honeymoon phase in these patients [49]. However, at least in non-diabetic adults, the contribution of ADSCs to beta cell mass is marginal, as hematopoietic stem cells derived from adult donors are not a source of pancreatic beta cells in adult non-diabetic humans [87]. Studies on AHSC in T1DM are shown in Table 4.

However, it is important to note that the side effects of the immunologic protocols include, but are not limited to, neutropenic fever, bone marrow suppression, and even death. Further investigation is warranted to determine whether using selected and better-characterized subsets of hematopoietic stem cells may provide greater benefits with fewer risks.

Another type of stem cells, ESCs are derived from the inner cell mass of blastocysts and have the capacity for extensive self-renewal, as well as the ability to differentiate into cell types from all three germ layers. In contrast, iPSCs are created by reprogramming somatic cells, such as peripheral blood mononuclear cells, dermal fibroblasts or adipocytes, back into a pluripotent state using specific factors. Pioneering research in 2014 successfully derived glucose-responsive, insulin-secreting cells that were capable of improving glucose control when transplanted into mice, and later in humans. As a result, ESCs and iPSCs can be produced in reasonable quantities and subsequently used to generate pancreatic endocrine cells by mimicking the molecular mechanisms that regulate pancreatic development in vivo, and first clinical experiences are reported [86]. The first protocols were developed more than ten years ago and have been tested in vitro and in animal models with encouraging results [88,89,90].

Immune rejection is a great challenge to be overcome in transplanting these type of cells, so several approaches have been tested to overcome it. Techniques such as cell encapsulation have been explored to create a barrier that protects the recipient from invading donor cells to a certain extent while safeguarding the donor cells from infiltrating immune cells. Additionally, these devices can physically restrict off-target cells and, when placed in appropriate locations, offer additional advantages, such as being accessible for environmental modifications and retrievable for analysis or safety assessments. Modified perforated encapsulation has been shown to allow for capillary ingrowth, enhancing the supply of nutrients and oxygen, which increases beta cell survival. However, this also exposes the β cells to the host immune system, necessitating the continued use of immunosuppression protocols to prevent immune-mediated rejection [91]. Several relatively small clinical trials have been published, but despite some promising results, insulin independence was not achieved in any of the study participants.

The first multicenter, open-label clinical study evaluating the safety, tolerability, and efficacy of pancreatic endoderm cells derived from human ESCs in macroencapsulated devices implanted subcutaneously was published in 2018. These cells were delivered in encapsulation devices designed to protect them from host-versus-graft reactions using a cell-impermeable layer. However, cell survival was inconsistent, and no clinically relevant insulin secretion was observed after a two-year follow-up. Additionally, the devices triggered a foreign body response in the host [92]. A subsequent paper on human EPC-derived pancreatic endoderm cells transplanted in patients with type T1DM described a study group of 17 patients participating in a Phase 1/2 clinical trial. All participants received pluripotent stem cell-derived pancreatic endoderm cells within a macroencapsulation device implanted subcutaneously, along with immunosuppression. In most patients, engraftment and insulin production were observed, with approximately one-third of the group demonstrating positive circulating C-peptide levels six months after the procedure. These initial data suggested that stem cell-based approaches may offer a scalable and renewable alternative to pancreatic islet transplants [93].

In a similar Phase 1/2 clinical trial involving patients T1DM, a non-immunoprotective macroencapsulation device containing human EPC-derived pancreatic endoderm cells was implanted subcutaneously in 15 individuals, alongside an immunosuppression regimen. These patients were followed for one year. The implants were well tolerated, with no severe graft-related adverse events. After implantation, patients exhibited increases in both fasting and glucose-responsive C-peptide levels, as well as mixed meal-stimulated C-peptide secretion. Explanted grafts showed cells with a mature β-cell phenotype that were immunoreactive for insulin, islet amyloid polypeptide, and MAF bZIP transcription factor A (MafA). Although no patient achieved insulin independence, better glucose regulation was observed, with reduced insulin requirements [94].

Two years later, in a follow-up clinical trial, 10 patients received two to three times more encapsulation devices containing more human EPC-derived pancreatic precursor cells implanted subcutaneously compared to previous studies, aiming to enhance the therapeutic effect. The immunosuppressive protocol included anti-thymocyte globulin for induction, followed by maintenance with mycophenolate mofetil and tacrolimus. By the 6th month, four patients exhibited detectable C-peptide levels, with clinically significant circulating C-peptide levels (greater than 0.1 nmol/L) observed in three patients. In these individuals, continuous glucose monitoring showed improved glucose regulation and decreased demand for exogenous insulin by the 9th month, and no severe hypoglycemic events. The single patient who developed detectable, but clinically insignificant C-peptide levels showed no improvements in glucose regulation [91].

Recently, a manuscript was published detailing a one-year follow-up of a single patient who received autologous iPSC-derived islets implanted subcutaneously, accompanied by an immunosuppressive treatment protocol. The patient, who had T1DM, achieved insulin independence approximately two and a half months after the islets were implanted, with improved glucose regulation parameters, such as HbA1c and time in range. At the one-year mark, the time in range exceeded 98% [73].

Considering directions for future research, the ideal beta cell therapy would not only generate an satisfactory supply of functional beta cells but also avoid immune reactions, including autoimmunity and tissue rejection. Consequently, the ultimate goal for the widespread clinical use of stem cell-derived islets is to bioengineer hypoimmunogenic cells capable of evading the immune response. This approach would eliminate the need for long-term or lifelong immunosuppressive therapy while ensuring adequate insulin secretion [95,96].

## 5. Ethical Considerations in Pancreas and Pancreatic Islet Transplantation, and Stem Cell-Base Therapies

Advances in transplantation and genetic technologies offer significant therapeutic potential for patients with T1DM. Still, they raise complex ethical considerations that must balance scientific progress with moral and societal values. These interventions demand robust ethical oversight, transparent patient education, and equitable access to ensure fair and ethical implementation. Pancreas transplantation involves ethical concerns surrounding donor organ allocation and patient selection. The scarcity of donor organs on one hand, and wider indications with broader patient selection for transplantation, necessitate stringent prioritization, raising questions about fairness and equity in access. Furthermore, pancreas transplantation is still widely restricted to cadavers, although transplantations from living donors are mentioned in the literature [97]. Transplantation involving living donors raises questions about the particular relationship between the recipient and the donor, with a particular focus on the potential for donor exploitation. Additionally, the long-term need for immunosuppressive drugs after transplantation poses significant risks, such as increased susceptibility to infections and other complications. Patients must fully understand and consent to these long-term implications [98].

Although pancreatic islet transplantation is less invasive than full organ transplantation, ethical concerns remain. These include using multiple donor pancreata as islet sources, recipient selection and the requirement for immunosuppressive drugs. Equity in access to these therapies is also a pressing issue. In addition, the risk of graft failure and the need for additional transplantations must be addressed. Therefore, the patient needs to be fully informed about the benefits and risks associated with the procedure. Ideally, these procedures should be provided first and foremost to patients who stand to benefit the most [99].

On the other hand, stem cell transplantation offers the potential to overcome some donor-related ethical concerns, as cells can often be harvested from embryos, fetuses, living donors or even the patients themselves, theoretically offering broader availability. However, sourcing heterologous stem cells, particularly embryonic and fetal stem cells, raises ethical issues. In particular, the ethical dilemma surrounding the destruction of human embryos has been, and continues to be, a significant factor slowing the development of hESC-based clinical therapies. Similarly, fetal stem cells, often derived from aborted fetuses or miscarriages, raise ethical concerns about the moral status of the fetus and the acceptability of using these cells in research and therapy.

The development of autologous stem cell transplantation and the use of iPSCs have largely addressed this ethical concern. However, significant challenges persist in the clinical translation of these methods. The unlimited differentiation potential of stem cells, coupled with the risks of unintended differentiation and malignant transformation, continues to pose major safety concerns. The same applies to the risks associated with immunosuppressive protocols [100].

Therefore, collaboration among ethicists, clinicians, and policymakers is essential to navigating the moral landscape of these interventions. Transparency about the cell sources, robust informed consent processes, and adherence to strict regulations are critical to mitigating these concerns. Striking a balance between fostering innovation, minimizing harm, respecting human dignity, and preventing misuse will be crucial to ensuring these technologies benefit all patients equitably [101].

## 6. Key Limitations of Published Studies

Although PTx, IT, and stem cell-based therapies for T1DM have been performed globally for a substantial period, and considerable experience and evidence have been gathered, several limitations exist in the papers included in this review.

The main limitation is the relatively small sample sizes, which reflect the stringent criteria for these treatments. Despite the small sample sizes, a wide range of findings has emerged from the manuscripts included in this review, depending on the study goals. These findings include various confounding factors affecting insulin independence and the impact of these therapies on other organs, such as the heart. Given the small sample sizes, a significant issue is the loss of study participants to follow-up, as missing data can considerably impact the study findings.

In some studies, particularly those involving newly diagnosed patients with T1DM, the inclusion of a control group receiving standard treatment could enhance the validity of the findings. This is important because the study treatment may interfere with the naturally occurring honeymoon period and the associated temporary insulin independence in these patients. However, in cohort studies reporting on patients with long-standing T1DM, a control group is not necessary when analyzing insulin independence, as this outcome cannot be achieved without some form of transplantation therapy.

Studies reporting data from large registries face specific issues, such as data protection regulations, including patient consent and other legal considerations for using this data for scientific purposes. Although unlikely, these issues may hinder data quality, in addition to the challenges posed by the retrospective design of such studies.

Furthermore, the reporting of insulin independence across studies was inconsistent and varied significantly depending on study aims and protocols. Some studies reported on short-term insulin independence, while others focused on sustained insulin independence, reflecting a lack of consensus in this area. Additionally, other relevant outcomes of transplantation therapies for T1DM include decreased insulin requirements, reduced glucose variability, increased time in range as detected by continuous glucose monitoring, and a decreased rate of microvascular and macrovascular complications. However, these outcomes were not consistently reported in the selected studies.

## 7. Conclusions

PTx and IT are procedures aimed at restoring insulin production and improving blood glucose management in individuals with brittle diabetes. PTx involves the surgical replacement of pancreatic function with a cadaveric pancreas. This procedure can be performed as a standalone operation or in conjunction with the transplantation of other organs, most commonly the kidney. IT involves isolating insulin-producing islet cells from a donor pancreas and infusing them into the recipient’s body, primarily into the liver. This procedure is less invasive than a full pancreas transplant. The latest transplantation technique, AHSCT, seems to be reserved for patients with newly diagnosed T1DM, modifying the autoimmune response and thus avoiding further pancreatic islet deterioration. The benefits of all three types of transplantation include improved blood glucose control and a reduced need for exogenous insulin, with insulin independence as the ideal outcome. This outcome is more likely to be achieved in patients undergoing Tx compared to those undergoing other transplantation methods.

Despite their advantages, these procedures present several challenges. The risk of rejection of the transplanted allogenic tissues necessitates immunosuppressive therapy, which carries risks of infections and other side effects. Surgical risks, such as infection and bleeding, are also concerns in pancreas transplantation. Furthermore, the limited availability of suitable donor pancreases can lead to long waiting times for candidates. In addition, AHSCT involves aggressive procedures necessary for stem cell harvesting, and the accompanying immunosuppression protocols carry substantial risks of complications.

Ongoing research in PTx and IT focuses on improving the success rates and safety of these procedures. Key areas of investigation include the exploration of regenerative medicine to generate insulin-producing cells from stem cells, the development of improved immunosuppressive protocols and immunoprotective devices that minimize side effects, and the investigation of anti-inflammatory therapies to protect islet cells during and after transplantation. Furthermore, this field of research could benefit from a broad consensus on success criteria and standardized reporting, which would allow for more concise comparisons among different studies.

## Figures and Tables

**Table 1:** Manuscript Quality Assessment Results.

	CASP Question:												
Author/Year	1	2	3	4	5	6	7	8	9	10	11	12	Main Limitations:
Marfil Gaza, 2023 [12]	+	+	+	+	+	+	+	+	+	+	+	+	retrospective design
Chetboun, 2023 [13]	+	+/−	+	+	+	+	+	+	+	+	+	+	retrospective design data from voluntary registry T1DM duration not reported
Hering, 2023 [14]	+	+/−	+	+	+	+	+	+	+	+	+	+	data from voluntary registry
Rickels, 2022 [15]	+	+	+	+	+	+/−	+	+	+	+/−	+/−	+/−	substantial part of participants lost to follow up relatively small sample size
Boggi, 2022 [16]	+	+	+	+	+	+	+	+	+	+	+	+	single center study relatively small sample size
Marfil Gaza, 2022 [17]	+	+	+	+	+	+	+	+	+	+	+	+	single center study retrospective design
LaBlanche, 2021 [18]	+	+	+	+	+	+/−	+	+	+	+	+	+	retrospective design small sample size substantial part of participants lost to follow up
Anteby, 2021 [19]	+	+	+	+	+	+	+	+	+	+	+	+	small sample size
Vantyghem, 2019 [20]	+	+	+	+	+	+	+	+	+	+	+	+	small sample size
Gu, 2018 [21]	+	+	+	+	+	+/−	+	+	+/−	+	+	+	small sample size substantial part of participants lost to follow up
Voglova, 2017 [22]	+	+	+	+	+	+	+	+	+	+	+	+	relatively small sample size single center study
Schive, 2017 [23]	+	+	+	+	+	+	+	+	+	-	+/−	+	retrospective design small sample size
Malmegrim, 2017 [24]	+	+	+	+	+	+	+	+	+	+	+	+	small sample size
Delaune, 2016 [25]	+	+/−	+	+	+/−	+	+	+	+/−	+	+	+	small sample size T1DM duration not reported
Moassesfar, 2016 [26]	+	+	+	+	+	+	+	+	+	+	+	+	small sample size
Hering, 2016 [27]	+	+	+	+	+	+	+	+	+	+	+	+	small sample size
Nijhoff, 2016 [28]	+	+	+	+	+	+	+	+	+	+	+	+	small sample size
Cantu Rodriguez, 2016 [29]	+	+/−	+/−	+	+	+	+	+	+/−	+	+	+	small sample size variable T1DM duration no control group
LaBlanche, 2015 [30]	+	+	+	+	+	+	+	+	+	+	+	+	relatively small sample size retrospective design
Lehmann, 2015 [31]	+	+	+	+	+	+	+	+	+	+	+	+	relatively small study group
Anazawa, 2014 [32]	+	+/−	+/−	+	+	+	+	+	+/−	+	+	+	small sample size T1DM duration not reported
D’Addio, 2014 [33]	+	+/−	+	+	+	+	+	+	+	+	+	+	no control group relatively small sample size
O’Connell, 2013 [34]	+	+/−	+	+	+	+	+	+	+	+	+	+	small sample size
Danielson, 2013 [35]	+	+	+	+	+	+	+	+	+	+	+	+	small sample size
Barton, 2012 [36]	+	+/−	+	+	+	+	+	+	+	+	+	+	retrospective design
Hirsch, 2012 [37]	+	+	+	+	+	+	+	+	+	+	+	+	small sample size
Bellin, 2012 [38]	+	+/−	+	+	+	+	+	+	+	+	+	+	retrospective design data from voluntary registry T1DM duration not reported
Gu, 2012 [39]	+	+/−	+	+	+	+	+	+	+	+	+	+	small sample size no control group
Borot, 2011 [40]	+	+	+	+	+	+	+	+	+	+	+	+	small sample size
Socci, 2010 [41]	+	+	+	+	+	+	+	+	+	+	+	+	small sample size
Kave, 2010 [42]	+	+	+	+	+	+	+	+	+	+	+	+	relatively small sample size single center study
Niclauss, 2011 [43]	+	+	+	+	+	+	+	+	+	+	+	+	relatively small sample size
Couri, 2009 [44]	+	+/−	+	+	+	+	+	+	+	+	+	+	small sample size no control group
Barton, 2007 [45]	+	+/−	+	+	+	+	+	+	+	+	+	+	retrospective design data from voluntary registry
Vantyghem, 2009 [46]	+	+	+	+	+	+	+	+	+	+	+	+	small sample size
Gerber, 2008 [47]	+	+	+	+	+	+	+	+	+	+	+	+	small sample size
Dieterle, 2007 [48]	+	+	+	+	+	+	+	+	+	+	+	+	small sample size
Voltarelli, 2007 [49]	+	+/−	+	+	+	+	+	+	+	+	+	+	no control group small sample size
Keymeulen, 2006 [50]	+	+/−	+	+	+	+	+	+	+	+	+	+	small sample size T1DM duration not reported
Shapiro, 2006 [51]	+	+	+	+	+	+	+	+	+	+	+	+	small sample size
Maffi, 2005 [52]	+	+	+	+	+	+	+	+	+	+	+	+	small sample size
Ryan, 2005 [53]	+	+	+	+	+	+	+	+	+	+	+	+	relatively small sample size
Michalak, 2005 [54]	+	+	+	+	+	+	+	+	+	+	+	+	relatively small sample size
Boggi, 2005 [55]	+	+	+	+	+	+	+	+	+	+	+	+	small sample size
Boggi, 2005 [56]	+	+/−	+	+	+	+	+	+	+	+	+	+	small sample size
Fiorina, 2000 [57]	+	+	+	+	+	+	+	+	+	+	+	+	small sample size
Secchi, 1997 [58]	+	+	+	+	+	+	+	+	+	+	+	+	small sample size
Kinkhabwala, 1996 [59]	+	+	+	+	+	+	+	+	+	+	+	+	small sample size

**Table 2:** Insulin independence rates, and study characteristics of PTx studies.

Author/Year	Study Population/Follow Up Period	Major Findings on Insulin Independence
Marfil Gaza, 2023 [12]	146 adults undergoing PTx median follow up period 7.4 years T1DM duration > 5 years	insulin independence occurred in 92.5% PTx recipients mean duration of insulin independence 6.7 (IQR 2.9–12.4) years
Chetboun, 2023 [13]	209 adults undergoing PTx (SPK 172, PTA and PAK 37) 5 year follow up T1DM duration not reported	186 (89%) patients were insulin independent after 5 years.
Boggi, 2022 [16]	66 adults undergoing PTA follow up period 10 years T1DM duration > 3 years	insulin independence or minimal insulin requirement was observed in 57.4% and 3.2% of patients, respectively.
Voglova, 2017 [22]	49 adults undergoing PTx (36 PTA,13 PAK) follow up period 5 years T1DM duration 16.5–31 years	insulin independence was observed in 73% of patients at one year, 68% at two years, and 55% at five years post-transplantation
Moassesfar, 2016 [26]	15 adults undergoing received PTA follow up period 12–118 months T1DM duration 29.9 ± 8.12 years	insulin independence was reported in 93% of patients after 1 year, and in 64% after 3 years of follow up mean duration of insulin independence was 55 months
Lehmann, 2015 [31]	94 adults undergoing SPK/PAK follow up period up to 13 years T1DM duration 32.1 ± 8.2 years	insulin independence was observed in 73.5% of patients five years post-transplantation
Bellin, 2012 [38]	677 adults with T1DM undergoing PTA follow up period 5 years T1DM duration not reported	insulin independence was observed in 52% of patients
Socci, 2010 [41]	17 adults with T1DM undergoing PTX (SPK 9, PTA 6, PAK 2). mean follow up period 37.1 months pediatric donors	insulin independence was observed in 82.35% of patients at 3 months, 69.68% at 6 months, and 63.35% at 1 year, respectively
Kave, 2010 [42]	68 adults undergoing SPK, receiving either systemic-bladder (SB) or portal-enteric (PE) drainage follow up period 2 years T1DM duration: 24.0 ± 6.3 and 26.5 ± 5.5 years for the SB and PE group	insulin independence was 77.9 ± 6.9% for the SB group and 81.3 ± 7.5% for the PE group at 1 year insulin independence was 77.9 ± 6.4% for the SB group and 71.4 ± 9.3% for the PE group at 2 years
Gerber, 2008 [47]	25 adults undergoing of SPK mean follow-up period 38 months T1DM duration: 30.3 ± 7.1 years	insulin independence was observed in 96% of patients after 1 year
Dieterle, 2007 [48]	38 adults with insulin independence at least 10 years after SPK follow up period 10 years T1DM duration 25 ± 1 years at SPK	insulin independence was observed in 28% of patients
Michalak, 2005 [54]	51 adults undergoing SPK follow up period 6–180 months T1DM 23 ± 4 years duration	insulin independence is observed in 68.5% of patients
Boggi, 2005 [55]	40 adults undergoing PTA mean follow-up of 16.4 months T1DM duration 23.9 ± 10.5 years	insulin independence is observed in 94.9% of patients
Boggi, 2005 [56]	16 adults undergoing PTA (6 SPK, 10 PTA) mean follow-up period of 26.6 months donor age > 45 years	insulin independence was observed in 81.2% and 67.7% after 1 and 5 years, respectively
Fiorina, 2000 [57]	42 adults undergoing SPK follow up period of 42 months T1DM duration 24 ± 1 years	insulin independence was observed in 100% of patients
Kinkhabwala, 1996 [59]	19 adults undergoing PTx (18 SPK, 1 PAK) mean follow-up period of 396 days mean T1DM duration 25 years	insulin independence was observed in 89% of patients

**Table 3:** Insulin independence rates, and study characteristics of IT studies.

Author/Year	Study Population/Follow-Up Period	Major Findings on Insulin Independence
Marfil Gaza, 2023 [12]	266 adults undergoing IT 20 year follow up duration of T1DM > 5 years	insulin independence occurred in 78.6% in IT recipients mean duration of insulin independence was 2.1 (IQR 0.8–4.6) for IT recipients
Chetboun, 2023 [13]	1210 adults undergoing ITA and IAK 5 year follow up T1DM duration not reported	insulin independence reported in 23.5% after 5 years
Hering, 2023 [14]	398 adults undergoing ITA follow up 5 years T1DM duration 30 ± 11 years	53% of patients were insulin-independent 105 patients received a single IT, 196 2 IT, and 97 3 IT
Rickels, 2022 [15]	72 adults undergoing ITx (48 ITA, 24 IAK) median follow up period 65.8 months (ITA) and 39.3 months (ITK) T1DM duration 31.5 ± 11.0 years (ITA) and 37.0 ± 10.0 years (IAK)	insulin independence was achieved by 74% of ITA and IAK transplantation recipients 57% of recipients maintained insulin independence during long-term follow-up 5 patients needed multiple procedures
Marfil Gaza, 2022 [17]	255 adults undergoing IT median follow up 7.4 years T1DM duration > 5 years	insulin independence was reported in 79% of patients, with a Kaplan–Meier estimate of 61% (95% CI 54–67) at 1 year, 32% (25–39) at 5 years, 20% (14–27) at 10 years, 11% (6–18) at 15 years, and 8% (2–17) at 20 years
LaBlanche, 2021 [18]	31 patients (13 IAK, 14 ITA, 4 lost to follow up) 10 year follow-up T1DM duration > 5 years	insulin independence was observed in 4.8% of patients patients usually received multiple infusions
Anteby, 2021 [19]	12 adults undergoing ITA median follow up period 56 months median T1DM duration 31.5 years	insulin independence was observed in 33% of patients at last follow up 8 patients required 2 IT procedures
Vantyghem, 2019 [20]	28 adults undergoing IT (14 ITA, 14 IAK) 10 year follow-up T1DM duration > 5 years	insulin independence was observed in 39% and 28% of patients 5 and 10 years after IT all patients received 2–3 islet infusions
Voglova, 2017 [22]	30 adult patients undergoing IT (24 ITA, 4 IAK, 2 SIK) 2 year follow up median T1DM duration 27.5	17% of IT recipients were temporarily insulin-independent. 10 patients had a single ITX procedure, 9 had 2 IT procedures and 11 had 3 procedures
Schive, 2017 [23]	18 adult patients undergoing ITA mean follow up 32.2 ±17.4 months mean T1DM duration 38, 26 and 47 years, respectively, in the single ITA, multiple ITA and ITA post PTX group	insulin independence was observed in 0% of patients after single ITA, in 20% after multiple ITA, and in 20% of patients after single ITA post PTx 3 patient had a single, IT, 10 patients multiple ITx procedures and 5 patients received ITx after PTx
Delaune, 2016 [25]	25 adult patients (14 ITA, AIK 7, SIK 4) mean follow up period 30.7 months T1DM duration unreported	76% of patients reached insulin independence at least at one point during follow up
Hering, 2016 [27]	48 adults undergoing ITA follow up period for 1 year median T1DM duration 28.5 years	insulin independence was reported in 52.1% at day 365 22 subjects received one islet infusion, 25 subjects received two infusions, and 1 subject received three infusions.
Nijhoff, 2016 [28]	13 adults undergoing IAK 2 year follow up T1DM duration 35 ± 9 years	the reported one- and 2-year insulin independence was 62% and 42%, respectively 13 patients received 22 IT in total
LaBlanche, 2015 [30]	44 adult patients undergoing ITX (24 ITA and 20 IAK) follow up period 60 month mean T1DM duration 33 years	in the IAK group, 45%, 40%, 40%, 35%, and 31.5% met the criteria of insulin independence at 12, 24, 36, 48, and 60 months, respectively. in the IAT recipients, 37.5%, 45.8%, 37.5%, 25%, and 14% met the criteria of insulin independence at 12, 24, 36, 48, and 60 months, respectively. 32 patients received two infusions, and 4 patients received three infusions
Lehmann, 2015 [31]	38 adults undergoing SIK/IAK transplantation follow up period up to 13 years T1DM duration 37.0 ± 11.0 years	the 5-year insulin independence rate was 9.3%
Anazawa, 2014 [32]	18 patients undergoing ITA follow-up period 76.4 ± 3.3 months T1DM duration not reported	3 patients achieved insulin independence for 14, 79 and 215 days 8, 4, and 6 recipients received 1, 2, and 3 islet infusions
O’Connell, 2013 [34]	17 adults underwent ITA 1 year follow up T1DM duration not reported	9 patients achieved insulin independence at study end 8 patients required 2–3 ITA procedures to become insulin independent
Danielson, 2013 [35]	15 adults undergoing ITA 1–5 year follow up T1DM duration 28.7 years	reported insulin independence rate of 100% after 1–3 transplants
Barton, 2012 [36]	677 adult undergoing ITA or IAK follow up 3 years T1DM duration 27.3, 29.6 and 31.4 years according to reported era	reported insulin independence at 3 years after IT were 27% in the early era (1999–2002 to 37% in the mid (2003–2006) and to 44% in the most recent era (2007–2010) 36% of the recipients received one infusion, 44% received two, 18% received three, 1.3% received four, and one person received six infusions
Hirsch, 2012 [37]	30 adults undergoing ITx (24 ITA, 6 IAK) mean follow-up 59.9 ± 22 months T1DM duration > 9 years	25 patients achieved insulin independence, 16 remained insulin independent > 1 year 1 patient received a single infusion, 19 patients 2 infusions, 8 patients 3 infusions, and 2 patients 4 infusions
Bellin, 2012 [38]	263 adults undergoing IT with different immunosuppression protocols follow up 5 years	reported insulin independence rate was 50% in those receiving anti-CD3 antibodies alone or T cell depleting antibodies and TNF-a inhibition
Borot, 2011 [40]	15 adults received IAK 2 year follow up mean T1DM duration 33 years	five of eight single-graft subjects (62.5%) became insulin independent versus five of seven in double-graft subjects (71.4%), five patients received a single infusion, others two
Niclauss, 2011 [43]	56 adults receiving ITA 1 year follow up mean T1DM > 30 years	insulin independence rate of 40% observed after one year follow up some patients required up to 3 ITx procedures
Barton, 2007 [45]	315 adults receiving ITx (285 IA, 30 IAK) 4 year follow up T1DM duration 18.7 ± 1.0 years	insulin independence was reported in 56% of patients 1 year after IT, and in 12% of patients 4 years after IT repetitive IT infusion boost the rate of insulin independence in the short term, with a decline afterwards to levels characteristic for single IT recipients
Vantyghem, 2009 [46]	14 adults received ITA mean follow up 3.3 years T1DM duration > 5 years	eight patients (57%) remained insulin independent patients received up to three IT
Keymeulen, 2006 [50]	22 adult patients receiving ITA 1 year follow up T1DM duration not reported	10 patients insulin independent after 1 year, 5 of them with a single IT 13 patients received a second ITA after 2 months
Shapiro, 2006 [51]	36 adults receiving ITA 1 year follow up T1DM duration > 5 years	insulin independence rate 44% afer 1 year 11 subjects receiving 1 infusion, 9 receiving 2 infusions, and 16 receiving 3 infusions
Maffi, 2005 [52]	31 adults receiving IAK follow-up period 38 ± 4 months T1DM duration 27 ± 2 years	9/31 of patients became insulin independent 8 patients received a second IAK
Ryan, 2005 [53]	69 median follow-up period 35.5 months T1DM duration 27.1 ± 1.3 years	44 patients were considered to have completed the islet transplant with insulin independence on the short term reported insulin independence rate of 10% after 5 years of follow up 52 patients had two transplants, and 11 subjects had three transplants
Secchi, 1997 [58]	20 adults receiving ITA follow up period at least 12 months T1DM duration 25 ± 1 years	insulin independence was reported in seven patients for an interval of 21 ± 7 months, range 2–58 months) one patient received 2 ITA

**Table 4:** Insulin independence rates, and study characteristics of AHSC studies.

Author	Study Population	Major Findings
Gu, 2018 [21]	15 newly diagnosed T1DM patients received AHSC transplantation mean age: 18 ± 3.9 years 48-month follow-up period	14/20 patients (70%) in the AHSC group experienced complete remission lasting 1.5–48 (median 20) months. three patients were insulin independent during 48 months single procedure
Malmegrim, 2017 [24]	21 newly diagnosed T1DM patients were monitored after AHSC transplantation patients’ age range 12–35 years median follow-up of 78 (range 15–106) months	21 patients became insulin-independent, but resuming insulin after median of 43 (range 6–100) months. single procedure
Cantu Rodriguez, 2016 [29]	16 patients aged 8–17 years received AHSC median follow-up period 34 months T1DM duration: 2–92 months	insulin independence reported at 7 patients (44%) at study end single procedure
D’Addio, 2014 [33]	65 patients with new-onset T1DM received AHSC follow up period 48 months reported patients’age: 20.4 ± 5.5	59% achieved insulin independence within the first 6 months, and 32% remained insulin independent to study end median duration of insulin independence: 12 months (range 12–40 months) single procedure
Gu, 2012 [39]	28 patients with mean age 17.6± 3.7 years mean follow up period of 19.3 months newly diagnosed T1DM	insulin independence, was observed in 15 of 28 patients (53.6%) and lasted from 3 to 42 months (median 24 months) 8 (28.6%) maintained insulin independence to study end, mean duration of 23.8 ± 12.7 months (range 3–42 months) single treatment
Couri, 2009 [44]	23 patients with newly diagnosed T1DM (aged 13–31 years, mean 18.4 years) mean follow-up period 29.8 months	insulin independence was reported in all patients (12 continuosly, 8 transiently) mean time of sustained insulin independence: 31 months, range,14–52months mean time of sustained insulin independence: 17.7 months, range 6–47months single procedure
Voltarelli, 2007 [49]	15 patients with newly diagnosed type 1 DM without ketoacidosis age range 14–31 years mean follow up 18.8 months	14 patients became insulin independent (1 patient for 35 months, 4 patients for at least 21 months, 7 patients for at least 6 months; and 2 patients with late response were insulin independent for 1 and 5 months, respectively) single procedure
